# Latarjet with cortical button fixation is associated with an increase of the risk of recurrent dislocation compared to screw fixation

**DOI:** 10.1007/s00167-019-05815-6

**Published:** 2019-12-17

**Authors:** Alexandre Hardy, Vincent Sabatier, Bradley Schoch, Marie Vigan, Jean David Werthel, Study Investigators, Geoffroy Nourissat, Philippe Valenti, Jean Kany, Julien Deranlot, Thibaud Vaugeois, Nicolas Solignac, Philippe Hardy

**Affiliations:** 1 Clinique du Sport, Paris, France; 2 Clinique des Maussins, Paris, France; 3 Mayo Clinic, Jacksonville, FLA USA; 4 Unité de Recherche Clinique Paris Saclay, Hopital Ambroise Paré, Boulogne-Billancourt, France; 5 Hopital Ambroise Paré, Boulogne-Billancourt, France

**Keywords:** Latarjet, Shoulder, Arthroscopy, Instability, Sport, Buttons, Screws, Fixation

## Abstract

**Purpose:**

The purpose of this study was to compare the clinical results of the Latarjet procedure using two cortical buttons vs two screws. It was hypothesized that cortical button would result in similar rates of recurrent dislocations, but a lower rate of reoperation compared to screw fixation.

**Methods:**

A retrospective comparative case-cohort analysis was performed for all patients undergoing a Latarjet procedure for recurrent anterior glenohumeral instability. Patient demographics, number of dislocations prior surgery, arm dominance, shoulder hyperlaxity, level of sport, type of sport and ISIS score were collected. Shoulders were separated into two groups based on surgical fixation (screws vs cortical button). Postoperatively, shoulders were evaluated for recurrent dislocation, revision surgery, post-operative Walch–Duplay score, and the Simple shoulder test (SST). Two hundred and thirty-six patients were included in the screw fixation group (group A) and 72 in button fixation group (group B) and were evaluated at a mean follow-up of 3.4 ± 0.8 years. Demographics of the two groups were similar with the exception of operative side hand dominance, which was more common in group B [50 (69.4%) vs 128 (54.2%), *p* = 0.02].

**Results:**

Recurrent dislocation was significantly lower in Group A: 6 (2.5%) vs 6(8.3%) (*p* = 0.02). Reoperation was more common in group A [14 (5.9%) vs 0 (0%)]. At follow-up, Walch–Duplay scores and simple shoulder tests were similar in both groups.

**Conclusion:**

Button fixation for Latarjet showed higher rates of recurrent dislocation compared to screw fixation. However, the increased stability afforded by screw fixation needs to be weighed against the increased risk of reoperation for hardware prominence.

**Level of evidence:**

III.

## Introduction

The Latarjet procedure is a commonly used procedure for chronic anterior shoulder instability associated with critical glenoid bone loss (> 15%) or in patients with a high risk of recurrence [28]. The transferred coracoid is used to reconstruct the missing anterior glenoid while the conjoined tendon adds a sling effect that is especially effective in abduction-external rotation [1, 26]. This procedure can be performed either open or arthroscopically with similar functional outcomes [19]. Several types of fixation have been proposed to secure the coracoid process to the glenoid. These include one [17] or two metallic screws [1], bioabsorbable screws [4], metallic plates [14] and more recently one [7] or two cortical buttons [32]. Most studies report the results of one or two metallic screws, as this is how the technique was originally described [22]. Metallic screw fixation has shown satisfactory results at long-term follow-up [1, 18]. Bioabsorbable screws are no longer used due to high rates of osteolysis [4]. Despite the clinical effectiveness and the biomechanical strength of screws [31], hardware-related complications remain the most frequently reported complications after a Latarjet (up to 46% of cases) [10, 23]. Hardware complications include intra- and post-operative fracture of the coracoid bone block secondary to overtightening of the screw(s) (1.1–1.5%), avulsion, twisting or breakage of the screw, and mechanical impingement between the screws and the humeral head or subscapularis (11–12%) [10, 16].

With the recent development of arthroscopic techniques, the use of one [6, 7] or two cortical buttons [32] has been proposed. These allow the use of a posterior drill guide, which has several advantages. First, it helps to accurately place the medio-lateral position of the bone block. Second, it helps optimize the angle between the glenoid face and fixation device (alpha angle). An alpha angle below 10° has been associated with decreased risk of suprascapular nerve injury [20] and coracoid nonunion [29]. Third, a posterior guide allows drilling from posterior to anterior, thus eliminating the need for a portal medial to the coracoid process, which is known to place the brachial plexus and axillary nerve at risk of injury [32]. Boileau et al. [7] reported a 91% rate of graft fusion at 6 months post-operatively using a single cortical button with no hardware-related complications.

To our knowledge, only one study has compared screw and cortical button fixation following Latarjet. However, some of the authors of the aforementioned study also designed the implants studied and 70% of the patients were lost to follow-up [25]. The purpose of this study was to compare the clinical results of the Latarjet procedure using two cortical buttons vs two screws. It was hypothesized that cortical button would result in similar rates of recurrent dislocations, but a lower rate of reoperation compared to screw fixation.

## Materials and methods

All patients were contacted and written consent was obtained for participation in this study. A retrospective comparative case-cohort analysis was performed for all patients undergoing a Latarjet procedure for recurrent anterior glenohumeral instability between 2013 and 2015. All operations were performed in one of five institutions. Patients older than 18 years at the time of surgery with a minimum 2 years follow-up were included. Patients were excluded if they had additional shoulder pathology at the time of surgery including posterior or multidirectional instability, pathological involvement of the long head of the biceps, rotator cuff tear, or symptomatic acromioclavicular joint pathology. Patients were also excluded if they could not speak or read French. Patients eligible for the study were identified from a computerized database that contains all patients who underwent surgery for shoulder instability in five different institutions. Medical records of all the eligible patients were reviewed by three independent reviewers to collect the following data: patient demographics, number of dislocations prior to surgery, arm dominance, shoulder hyperlaxity, level of sport, type of sport and ISIS score. Shoulder hyperlaxity was defined as passive external rotation ≥ 85° or a Gagey test > 95° as previously described by Balg et al. [5]. An episode of dislocation was defined as a glenohumeral dislocation that required reduction by someone else. The level of sport was categorized as “competition”, “recreational” or “none”. Sport type was categorized as “contact/collision”, “throwing sports”, or “other”. Standard pre-operative anteroposterior radiographs of the shoulder were reviewed for a Hill–Sachs lesion visible in external rotation and loss of contour of the glenoid as described by Balg et al. [5].

Four hundred and thirty-one patients met inclusion criteria, 121 shoulders did not respond to attempted contact or refused participation. One additional patient was unable to answer the provided questions due to a language problem. Thus, 308 (73.3% [CI 95% = 68.4%; 78.3%]) shoulders were included for analysis.

### Surgical procedure

All surgeries were performed by fellowship-trained shoulder surgeons with an annual surgical volume of over 50 shoulder instability cases per year. Two different techniques were used depending on the surgeon’s choice and habits, with all surgeons having at least 2 years of experience using their chosen technique.

Group A: a mini-open technique using (1) an operation-specific drill guide (Arthrex, Naples FLA) and two 4-mm cannulated cancellous screws based on the surgical technique as described by Walch [33] or (2) an arthroscopic technique as described by Lafosse et al. [21] using a specific guide (DePuy Mitek, Raynham, MA) and two 3.5-mm cannulated cancellous screws.

Group B: an arthroscopic technique using two cortical buttons [TightRope (Arthrex, Naples)] placed through a custom-made posterior drill guide with a fixed 7 mm offset (Vims, Villeneuve-lès-Bouloc, France) with concurrent Bankart repair [32].

### Post-operative management

All patients followed a similar post-operative protocol and were placed in a sling for the first week post-operatively. At the beginning of the second week, patients were encouraged to start self-assisted rehabilitation for 3 weeks. At 1 month post-operatively, patients were referred to a physiotherapist to start active mobilization in elevation and external rotation.

### Assessment at latest follow-up

Following an initial chart review, all eligible patients were contacted via phone and mailed/emailed a questionnaire to assess shoulder function and instability. Patients were given 1 month to respond before being contacted again by a member of the study team via phone. The questionnaire assessed the number of episodes of dislocation before surgery, the time between the first dislocation and surgery, recurrent dislocation, reoperation, a Walch–Duplay score [33], a simple shoulder test (SST) [24], and a visual analogue scale (VAS) pain score. Recurrence was defined as a new episode of dislocation that required reduction by someone else.

Approval was obtained from the ethics committee of Maussins-Nollet clinic (Paris).

### Statistical analysis

Quantitative data were described as mean ± standard deviation. Qualitative data were presented as numbers and proportions. Time to recurrence was calculated from the date of surgery to the date of recurrence. Patients without a recurrent dislocation at the date of last follow-up were censored at this time. Recurrence was analysed using the Cox proportional hazard model. Hazard ratios and their associated 95% confidence intervals were calculated. The Wilcoxon rank-sum test was used to compare groups in the quantitative analysis, while the Fisher exact test was used in the qualitative analyses. Analyses were performed with SAS software, version 9.4 (SAS Institute, Cary, North Carolina). The limit of significance was defined as *p* < 0.05.

## Results

Two hundred and thirty-six patients were included in the screw fixation group (group A) and 72 in the button fixation group (group B). Demographics of the two groups were similar with the exception of operative side hand dominance which was more common in group B [50 (69.4%) vs 128 (54.2%), *p* = 0.02]. The number of prior dislocations was similar between Groups A and B (6.4 ± 10.1 vs 7.4 ± 13.8) (n.s.). Participation in contact sports was also similar between Groups A and B [103 (43.6%) vs 29 (40.3%)]. The average ISIS scores were similar (4.9 ± 2.0 vs 5.0 ± 1.8). See Table 1 for full demographic information.

**Table 1:** Patients’ demographics between screw and button fixation cohorts

Patient demographics	Group A (*N* = 236)	Group B (*N* = 72)	*p* value
Age at surgery (years), mean (std)	27.8 ± 9.2	27.9 ± 10.0	n.s
Sex, *n* (%)			n.s
Male	201 (85.2%)	62 (86.1%)	
Female	35 (14.8%)	10 (13.9%)	
BMI, mean (std)	24.4 ± 3.5	24.1 ± 3.2	n.s
Number of dislocations	6.4 ± 10.1	7.4 ± 13.8	n.s
Dominant side operated	128 (54.2%)	50 (69.4%)	**0.02**
Type of sport, *n* (%)			n.s
Contact forced ABER	32 (13.6%)	17 (23.6%)	
Contact	103 (43.6%)	29 (40.3%)	
Other	81 (34.3%)	21 (29.2%)	
No sport	20 (8.5%)	5 (6.9%)	
Level of sport, *n* (%)			n.s
Competition	75 (31.6%)	28 (38.9%)	
Recreational	142 (59.9%)	39 (54.2%)	
No sport	20 (8.4%)	5 (6.9%)	
Hyperlaxity	79 (33.5%)	26 (36.1%)	n.s
Hill–Sachs lesion	172 (73.5%)	46 (63.9%)	n.s
Glenoid defect	175 (74.8%)	57 (79.2%)	n.s
ISIS score, mean (std)	4.9 ± 2.0	5.0 ± 1.8	n.s
Time between first dislocation and surgery, mean (std)	37.8 ± 57.6	52.8 ± 85.0	n.s
Previous Bankart repair, *n* (%)	20 (8.5%)	4 (5.6%)	n.s
Delay after Bankart, mean (std)	6.6 ± 7.9	5.8 ± 4.9	n.s

Bold value represents the statistically significant value

Mean follow-up for the entire group was 3.4 ± 0.8 years [min; max = 2.1; 5.3] and was not significantly different between the two groups (3.4 ± 0.8 [min; max = 2.1; 5.1] vs 3.3 ± 0.9 [2.1; 5.3]) (n.s.). The recurrence rate was lower in Group A than Group B: 6 (2.5%) vs 6 (8.3%) (*p* = 0.02). Patients in Group B had a higher risk of recurrence (hazard ratio = 3.8 [IC 95% = 1.2; 11.7], *p* = 0.01) than patients in Group A. The mean time from Latarjet to recurrent dislocation was similar between Groups A and B (2.1 ± 0.9 years vs 3.1 ± 1.3 years) (n.s.). The low recurrence rate within Group A was similar in both open (52 shoulders, 1.9%) and arthroscopic (184 shoulders, 2.7%) techniques (n.s.).

No patients in Group B required a return to the operating room, compared to 14 (5.9%) in Group A. Hardware removal represented 57.2% (*n* = 8) of the indications for reoperation followed by arthrolysis (*n* = 3, 21.4%), Eden-Hybinette (*n* = 2, 14.3%) and hematoma (*n* = 1, 7.1%). The mean time between Latarjet and reoperation was 1.2 ± 1.1 years [0.05; 3.4], Table 2.

**Table 2:** Comparison of recurrence and reoperation rates between screw and button fixation cohorts

Recurrence and reoperation	Group A (*N* = 236)	Group B (*N* = 72)	*p* value
Recurrence, *n* (%)	6 (2.5%)	6 (8.3%)	0.02
Delay before recurrence (years), mean (std)	2.1 ± 0.9 [0.6; 4]	3.1 ± 1.3 [0.9; 3.6]	n.s
Reoperation, *n* (%)	14 (5.9%)	0 (0.0%)	0.046
Hardware removal	8 (57.2%)	0	
Arthrolysis	3 (21.4%)	0	
Hematoma	1 (7.1%)	0	
Eden-Hybinette	2 (14.3%)	0	
Delay before reoperation (years), mean (std)	1.2 ± 1.1 [0.05; 3.4]		

At follow-up, both groups demonstrated similar pain scores (1.3 ± 1.8 vs 1.3 ± 2.1) (n.s.), Walch–Duplay scores (70.4 ± 25.6 vs 71.1 ± 23.4) (n.s) and SST (10.6 ± 2.0 vs 10.6 ± 2.0) (n.s.).

## Discussion

Patients undergoing a Latarjet procedure with cortical button fixation demonstrated a significantly higher rate of recurrent instability than patients treated with screw fixation [6 (8.3%) vs 6 (2.5%), *p* = 0.02]. The first hypothesis was rejected, with cortical button fixation leading to an almost four times increased risk of recurrent dislocation compared to screw fixation.

Recurrent instability is the second most common complication following Latarjet, occurring at a rate of 6.0 ± 1.2% [10]. Using button fixation, Boileau et al. reported a 2.9% recurrence rate at 6 months [13] and 2% at 35 months [8]. This is less than the recurrence rate following button fixation in our cohort (8.3%) at similar follow-up of 3.4 years. Metais et al. compared screw and cortical button fixation, finding a higher rate of recurrent dislocation with button fixation (6.25% vs 3.5%) at a mean follow-up of 23 months [25]. However, this study was limited by a high rate of patients lost to follow-up. In both studies, cortical button fixation exceeded the recurrence rate reported in Butt et al.’s [10] systematic review (6%). This raises concern about the potential lower efficiency of the button fixation to stabilize the shoulder after a Latarjet procedure. One explanation could be that, in this study, the cortical buttons were tightened using manual strength and not a suture tensioner. However, the effect of button tension was not evaluated in that study, and its role in clinical failure cannot be determined in this cohort.

Despite a higher rate of recurrence, no reoperations were needed in the group treated with cortical button fixation, compared to 5.9% (*n* = 14) in the screw fixation group. Hardware complications have been reported to affect up to 46% of patients following Latarjet with screw fixation [23]. In a study of 83 shoulders treated with arthroscopic Latarjet, Athwal et al. [3] reported a 4% rate of hardware removal. In a systematic review, Griesser et al. [16] reported that symptomatic hardware was the primary cause for reoperation in 6.5% of the cases. This was secondary to fixation failure in 3.8% and bone/soft tissue irritation in 2.7%.

The suture button has been described as a more technically challenging procedure with a long learning curve [27]. Bonnevialle et al. [9] reported a higher rate of complications during the early portion of the learning curve of arthroscopic Latarjet with cortical button fixation. All complications occurred within the first ten procedures and operative time stabilized after 30 cases [12]. With 90% of bone blocks positioned flush to the face of the glenoid after 20 cases, this technique shows promising results. The rates of flush bone position reported by Bonnevialle et al. [9] are higher than the 70% flush position reported by Casabianca et al. [11] using arthroscopic screw fixation. Cortical buttons also have the theoretical advantage of increasing the bone contact surface area between the coracoid and glenoid neck, with only one or two 2.8-mm holes compared to the two classical 3.2-mm holes. In another series of 76 cortical buttons, Boileau et al. [6] reported no complications with an 83% graft union rate. In comparison, Randelli et al. [30] reported a 77.6% graft fusion rate with arthroscopic screw fixation and an 88.6% rate with open screws fixation. Due to the design of the present study, post-operative CT scans were not obtained for all patients, and the effect of graft healing on recurrent dislocation could not be assessed. Future studies with post-operative axial imaging are needed to further evaluate the failure mechanism of cortical button fixation after Latarjet.

One of the main concerns regarding double-button fixation is its biomechanical strength. Recent biomechanical studies have shown that 4.0 mm cannulated and 3.5 mm cortical screws demonstrate similar loads to failure following cyclic loading [2]. Further work by Shin et al. reported that unicortical fixation is also sufficient [32], and may help to reduce the risk of suprascapular nerve injury. Quality biomechanical comparisons of button and screw fixation remain absent from the scientific literature. Provencher et al. evaluated eight pairs of matched male cadavers using double-button and screw fixation. The tests were performed pulling on the conjoined tendon in a 60° ABD and 60° external rotation and showed similar ultimate loads to failure and mean strain at failure for both double-button and screw fixation [27]. However, the mechanism of testing should be questioned, as other biomechanical studies measure the strength of the fixation by placing the testing force onto the bone block and directed medially similar to a humeral head dislocation [2, 31]. This loading mechanism has not been yet tested for cortical button fixation. In an ankle syndesmosis repair study, Goetz et al. showed that double-button fixation was insufficient to control sagittal plane stress compared to screw fixation [15]. Even though it cannot be compared directly to the shoulder, this highlights the need for new biomechanical studies evaluating this type of fixation using physiologic loading conditions that can be expected during shoulder dislocation.

There were several limitations to this study. First, this was a retrospective cohort study with 76% follow-up. The results may have been affected by nonresponse bias, as it is possible that patients who had a stable and pain-free shoulder did not take time to answer the questionnaire. On the contrary, it is possible that patients who experienced post-operative instability and/or dissatisfaction after the index surgery were more likely to be lost to follow-up. Second, there exists the possibility of performance bias in surgical technique secondary to differing techniques across five fellowship-trained surgeons. Cortical button fixation remains less popular than traditional screw fixation, which led to an unequal distribution of patients between groups and introduces the possibility of selection bias. However, sampling across five fellowship-trained practices using different surgical techniques may better reflect general practice. Third, no post-operative axial imaging was available for review. CT evaluation may have provided better understanding for the high rate of recurrence in the button group.

This study shows that the performance of the Latarjet using cortical buttons is not equivalent to the gold standard fixation using two screws. Although cortical buttons allow a simplification and a standardization of the arthroscopic surgical technique with a potential lower rate of hardware prominence, it is associated with a significantly higher rate of recurrent dislocation.

## Conclusion

Button fixation for the Latarjet procedure showed higher rates of recurrent dislocation compared to screw fixation. However, despite lower rates of recurrent instability reoperations were more common following screw fixation.
